# Irisin as a Potential Biomarker Associated with Myocardial Injuries in Patients with Severe Hypothyroidism

**DOI:** 10.1155/2021/3116068

**Published:** 2021-11-18

**Authors:** Zhi Yao, Xiaoyu Ding, Xia Gao, Ning Yang, Yumei Jia, Jia Liu, Guang Wang

**Affiliations:** Department of Endocrinology, Beijing Chaoyang Hospital, Capital Medical University, Beijing 100020, China

## Abstract

**Objective:**

Irisin, a novel myokine, has recently been considered to produce a cardioprotective effect. Potential biomarkers for myocardial injuries in patients with severe hypothyroidism have yet to be identified. We aimed to investigate whether serum irisin may serve as a promising biomarker for early detecting the myocardial injuries in patients with severe hypothyroidism.

**Methods:**

This cross-sectional study comprised 25 newly diagnosed drug-naive patients with severe primary hypothyroidism and 17 age- and sex-matched healthy controls. Circulating irisin levels and cardiac magnetic resonance (CMR) were evaluated in each participant. Left ventricular (LV) myocardial injuries were detected by CMR-based T1 mapping technique using a modified look-locker inversion recovery (MOLLI) sequence, which is quantified as native T1 values.

**Results:**

Compared with healthy controls, the severe hypothyroidism group had significantly lower levels of serum irisin, especially those with pericardial effusion (*P* < 0.05). The severe hypothyroidism subjects exhibited lower peak filling rates (PFRs) and higher native myocardial T1 values than controls (*P* < 0.05). Additionally, the ROC analysis displayed that the sensitivity and specificity of serum irisin for diagnosing pericardial effusion in patients with severe hypothyroidism were 73.3% and 100.0%, respectively. The AUC was 0.920 (0.861–1.000) (*P* < 0.001). The cutoff value was 36.94 ng/mL. Moreover, the results in subgroup analysis revealed that the native T1 values of the low-irisin group were significantly higher than that of the high-irisin group (*P* < 0.05). According to multivariate linear regression analysis, serum irisin concentrations were negatively and independently correlated with native myocardial T1 values after adjustment for age, sex, and other conventional confounding factors (*β* = −1.473, *P* < 0.05).

**Conclusions:**

Irisin may be a potential biomarker for predicting myocardial injuries in patients with severe hypothyroidism.

## 1. Introduction

Irisin, a novel myokine, is cleaved from the transmembrane protein fibronectin type III domain-containing protein 5 (FNDC5), the secretion of which is increased in response to exercise [1]. It has gained greater notoriety and visibility for its roles in regulating body metabolism and energy homeostasis by promoting the browning of white adipose tissue [2–6]. Additionally, this myokine has a high expression in the muscle, as well as heart and adipose tissues [4, 7], and circulating irisin mainly comes from heart and skeletal muscles [1, 8–10]. Many preclinical and clinical studies have been conducted to measure irisin and relate it to physiological or diseases conditions, such as metabolic and cardiovascular diseases [4, 10–12].

Thyroid hormones have long been known as a crucial regulator of basal metabolic rate and energy thermogenesis [13]. Impaired thyroid function has a negative impact on muscles, oxidative stress, metabolic regulation, and cardiac function [14–16]. As a result of the deficiency of thyroid hormones, the deposition of myocardial interstitial collagen and mucopolysaccharides (glycosaminoglycans) was believed to play a key role in the pathophysiological progress of myocardial fibrosis and edema in hypothyroidism [17, 18]. Our previous studies showed that diffuse myocardial injuries, measured by cardiac magnetic resonance (CMR)-based T1 mapping technique, were present in patients with overt hypothyroidism [19, 20]. Additionally, our group has also shown that serum irisin levels were declined in hypothyroidism subjects, and its levels might be restored after levothyroxine treatment [21].

Thus, thyroid dysfunction could directly or indirectly be associated with irisin modulation, or vice versa. Specially, there may be some potential links between circulating irisin concentration and myocardial damage in hypothyroidism. So far, very few studies have focused on their relationship among patients with severe hypothyroidism. We aimed to determine whether the serum irisin can serve as a potential biomarker associated with myocardial injuries in patients with severe hypothyroidism.

## 2. Materials and Methods

### 2.1. Study Population

A total of 25 newly diagnosed and untreated patients with severe primary hypothyroidism attending our outpatient clinic were enrolled in this cross-sectional study between December 2014 and September 2015. Meanwhile, 17 age- and sex-matched participants with no documented medical history were formed the healthy control group. The inclusion criteria were age between 18 and 65 years and serum thyroid-stimulating hormone (TSH) level > 50 *µ*IU/mL [22]. The exclusion criteria were the presence of known heart disease (coronary disease, myocarditis, valvular heart disease, heart failure, and so on), hypertension, diabetes, pregnancy, chronic obstructive pulmonary disease, liver or kidney failure, asthma, neoplastic disease, claustrophobia, or metal implants. This study protocol was conducted according to the Declaration of Helsinki ethical principles and approved by the Ethics Committee of our hospital. Each participant provided written informed consent.

### 2.2. Clinical and Biochemical Measurements

All subjects underwent anthropometric indicators, medical history, and biochemical tests. Data regarding height and body weight were recorded. After an overnight fast, blood samples were collected during 8:00–9:00 a.m. and instantly centrifuged at 3500 r/min for 10 min at 4°C, and then serum was separated and stored frozen at −80°C for biochemical and irisin assays. Total cholesterol (TC), high-density lipoprotein cholesterol (HDL-C), low-density lipoprotein cholesterol (LDL-C), triglyceride (TG), and creatine kinase (CK) were analyzed using a Dade Behring Dimension RXL autoanalyzer (Dade Behring Diagnostics, Marburg, Germany). Serum-free thyroxine and thyrotropin concentrations were measured by electrochemiluminescence immunoassay using Abbott Architect i2000 (Abbott Diagnostics, Abbott Park, IL, USA). Serum irisin levels were determined with enzyme-linked immunosorbent assay (ELISA) kits (Phoenix Pharmaceuticals, Inc., Burlingame, CA, USA). Body mass index (BMI) was derived as BMI (kg/m^2^) = body weight (kg)/height (m)^2^. Body surface area (BSA) was calculated as BSA (m)^2^ = 0.007184 × height (cm)^0.725^ × weight (kg)^0.425^.

### 2.3. Cardiac Magnetic Resonance Imaging

CMR studies were performed using a clinical 3T Tim Trio System scanner (Siemens Healthcare, Erlangen, Germany) in each participant. Modified look-locker inversion recovery (MOLLI) T1 maps [23] were obtained for assessing diffuse myocardial injuries. The detailed CMR protocol and image analysis are available in our previous studies [19, 20].

Based on each MRI, BSA-normalized left ventricular (LV) functional parameters were assessed according to the Society for CMR guidelines [24], including LV end-diastolic volume (EDV), LV end-systolic volume (ESV), LV mass (LVM), peak ejection time (PET), peak ejection rate (PER), peak filling time (PFT), peak filling rate (PFR), and heart rate (HR). The cardiac index (CI), stroke volume (SV), LVM index (LVMI), and ejection fraction (EF) were calculated by validated CMR-derived measures. Manual endo- and epicardium contours were drawn to measure a mean native T1 value of the mid-short axial slices. Meanwhile, the accompanying signs (pericardial effusion) were performed in each patient.

### 2.4. Statistical Analysis

Statistical analysis was done with SPSS software version 25.0 (IBM, Chicago, IL, USA) and GraphPad Prism 8.0 (Inc, CA, USA). Categorical variables were performed as frequencies or percentage. Continuous data were tested for normality using the Kolmogorov–Smirnov test. Normally distributed data were presented as means ± standard error of the mean (SEM), while variables with a skewed-distribution were expressed as median (interquartile range, IQR). The proportions were analyzed using chi-squared tests. The differences between two groups (control and severe hypothyroidism, two subgroups in the severe hypothyroidism group) were analyzed through an independent *t*-test for normally distributed variables or a Mann–Whitney *U* test for skewed-distribution variables. Multiple comparisons were performed by the one-way ANOVA or Kruskal–Wallis test followed by a *post hoc* test. Receiver operating characteristic (ROC) curve analysis was performed to assess the predictive power of irisin for determining pericardial effusion in patients with severe hypothyroidism and to identify an optimal cutoff value based on the balance between sensitivity and specificity. Correlations between irisin levels and native T1 values were analyzed by Pearson correlation coefficients. Parameters that show a significant correlation with irisin were subsequently calculated using a multivariate linear regression model after adjusting for potential confounding factors. A two-tailed *P*-value of <0.05 was considered statistically significant.

## 3. Results

### 3.1. Clinical Characteristics of the Control and Severe Hypothyroidism Groups

A total of 42 drug-naïve participants were enrolled in our study, including 25 subjects with severe hypothyroidism and 17 healthy controls.  Table 1 displays the clinical characteristics of all subjects. No difference was observed in HR and HDL-C levels between the two groups (Table 1). People with severe hypothyroidism had significantly higher BMI, TC, TG, LDL-C, CK, and TSH concentrations, while free T3 (FT3) and free T4 (FT4) levels were significantly reduced compared with controls (all *P* < 0.01). Of note, irisin concentrations were significantly lower in the severe hypothyroidism group than in controls (*P*=0.014).

### 3.2. Cardiac Magnetic Resonance Findings

CMR measurements of the study population are shown in Table 2. It was observed that 10 of the participants with severe hypothyroidism exhibited pericardial effusions. No significant differences were found in the majority of the CMR parameters between the two groups, including BSA, EF, SV, CI, PET, PFT, and PER, or any parameters of volumetric indexes such as EDV, ESV, and LVMI. However, the reduced PFR (*P* < 0.05) suggested early impairment of diastolic function in the severe hypothyroidism group. Furthermore, compared with the control group, native MOLLI (Table 2) showed a significant increase in myocardial T1 values among patients with severe hypothyroidism (*P* < 0.01). These results indicated the presence of early diffuse myocardial lesions in patients with severe hypothyroidism.

### 3.3. The Predict Value of Irisin for Pericardial Effusion in Subjects with Severe Hypothyroidism

**Figure 1:**
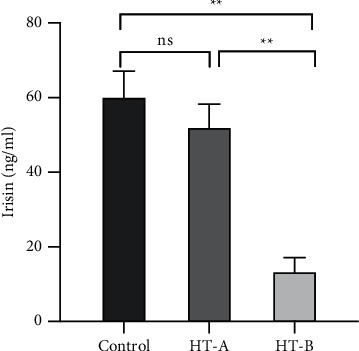
Mean serum irisin level in controls and severe hypothyroidism patients with (HT-B) or without (HT-A) pericardial effusion. ^*∗∗*^*P* < 0.01.

**Figure 2:**
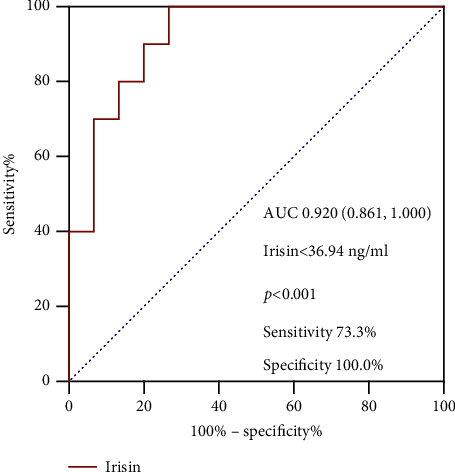
ROC curve analysis of irisin to predict pericardial effusion in severe hypothyroidism patients.

As mentioned above, subjects with severe hypothyroidism had significantly lower irisin concentrations than controls (Table 1). Moreover, circulating irisin levels were even lower in the severe hypothyroidism subgroup with pericardial effusion (13.16 ± 3.93 ng/mL) compared with those without pericardial effusion (51.85 ± 6.38 ng/mL) (*P* < 0.01). Nevertheless, there was no statistically significant difference in the levels of irisin between the severe hypothyroidism subgroup without pericardial effusion and the control group (Figure 1 and Supplementary Table 1). As expected, LV native T1 values were higher in the subgroup of severe hypothyroidism with pericardial effusion, indicating they had more severe diffuse myocardial injuries (*P* < 0.001, Supplementary Table 1). In order to identify the diagnostic value of serum irisin levels for pericardial effusion in severe hypothyroidism patients, we performed ROC analysis, as shown in Figure 2. The sensitivity and specificity of serum irisin in diagnosing pericardial effusion were 73.3% and 100.0%, respectively. The AUC was 0.920 (0.861–1.000) (*P* < 0.001). And the cutoff value of serum irisin for diagnosing pericardial effusion in patients with severe hypothyroidism was 36.94 ng/mL.

### 3.4. Relationship between Irisin Levels and Cardiac Magnetic Resonance Parameters

Next, we analyzed the relationships between CMR parameters and serum levels of irisin in subjects with severe hypothyroidism. According to the cutoff value of serum irisin, all severe hypothyroidism participants were divided into two groups: the high-irisin group and the low-irisin group (Table 3). Significantly, compared with the other group, the LV native T1 values were increased in the low-irisin subgroup (*P* < 0.05). However, no significant difference between the subgroups was observed in BSA, EDV, ESV, LVMI, EF, SV, CI, PET, PFT, PER, and PFR (Table 3).

**Figure 3:**
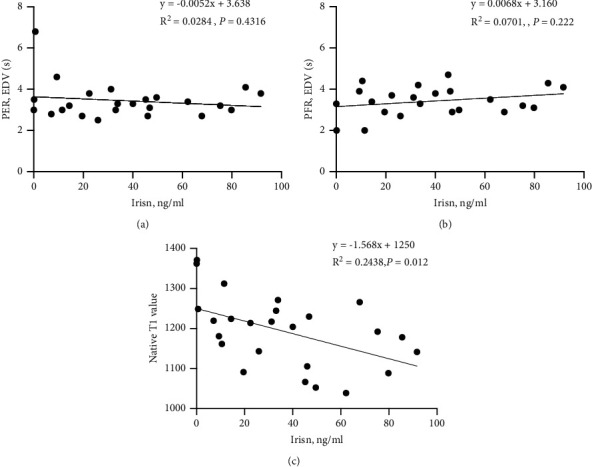
Correlation between serum irisin levels and PER (a), PFR (b), and native T1 values (c) in patients with severe hypothyroidism.

Bivariate correlation analyses were conducted to further determine the relationship between serum levels of irisin and CMR parameters among patients with severe hypothyroidism. We found that the circulating irisin concentrations were negatively correlated with LV T1 value levels (*r* = −0.494, *P* < 0.05, Figure 3). No correlation was observed between serum irisin and other CMR parameters. Collectively, our findings revealed that irisin levels were related to diffuse myocardial injuries.

### 3.5. Association between Irisin Levels and Myocardial Native T1 Values

To determine the role of irisin as an independent determinant of diffuse myocardial injuries in patients with severe hypothyroidism, we conducted multivariate linear regression to further examine the parameters that were considered clinically relevant or significant in univariate analyses (Table 4). Irisin levels were significantly associated with native T1 values (*β* = −1.568, *P* < 0.05) without adjustment of potential confounders. In model 1, with adjustment for age and sex, the relation between them was consistent (*β* = −1.617, *P* < 0.05). Interestingly, in model 2, with further adjustment with BMI, TG, TC, and FT4, we found that serum irisin levels remained inversely and independently associated with native T1 values in severe hypothyroidism patients (*β* = −1.473, *P* < 0.05). Taken together, irisin was an independent predictor for diffuse myocardial injuries in patients with severe hypothyroidism.

## 4. Discussion

The current study, for the first time, determined whether circulating irisin may serve as a promising predictive biomarker for early detecting myocardial injuries of severe hypothyroidism. Here, we found reductions in serum irisin concentrations and PFRs, as well as elevations in native T1 values in subjects with severe hypothyroidism. Serum irisin levels could distinguish severe hypothyroidism patients with from without pericardial effusion. Moreover, further analysis showed the LV native T1 values were significantly elevated in the low-irisin subgroup, and serum irisin levels were independently related to diffuse myocardial injuries (fibrosis and edema) in subjects with severe hypothyroidism.

Irisin, an exercise-induced myokine, is cleaved from the transmembrane protein FNDC5 before being released into the circulation and regulated by peroxisome-proliferator-activated receptor gamma (PPAR*γ*) coactivator 1-alpha (PG1-*α*) [1, 25]. As important components for regulating metabolism and thermogenesis, both irisin and thyroid hormones have profound functions [26]. To date, several studies have explored the connection between irisin and thyroid hormones, but the evidence is still contradictory. In people with clinical hypothyroidism, irisin concentrations have been depicted as either increased [27] or decreased [21, 28, 29]. We discovered that irisin levels were significantly reduced in the severe hypothyroidism group than in controls. Consistent with results shown in the current trial, a recent meta-analysis revealed that the levels of irisin are lower in hypothyroidism patients [30]. In contrast, a case-control research showed that circulating irisin concentrations were elevated in hypothyroidism [27]. There are several explanations for this contradiction. First, their study included participants with mild hypothyroidism (average TSH level, 13.1 mIU/mL). Second, irisin values measured with ELISA kits may vary widely among different assays [31, 32]. Animal studies also have confirmed the upregulation of serum irisin levels in hyperthyroid and hypothyroid rat models, which may be correlated with muscle damage observed in both conditions [26]. In this regard, previous data revealed that only prolonged hypothyroidism was interrelated with a significant decrease in irisin levels, which may be due to initial muscle destruction and leakage from damaged muscle cells and following a reduction in irisin production because of long-lasting myopathy [29].

In addition to regulating metabolism and thermogenesis, irisin is also considered to produce a cardioprotective effect in recent years [8, 33, 34]. Dun et al. first reported the high expression of irisin in cardiomyocytes [35]. Irisin can blunt collagen synthesis and myocardial fibrosis in angiotensin II-induced mice, and its mechanism may be to activate the Nrf2 pathway and inhibit the ROS/TGF-*β*/Smad signaling axis in cardiac fibroblasts [36]. Additionally, Liao et al. suggested that irisin treatment promoted angiogenesis via an ERK-dependent pathway and thus decreased cardiac fibrosis and ventricular dilation [37]. Furthermore, FNDC5 overexpression significantly improved obesity-induced myocardial inflammation, fibrosis, oxidative stress, and cardiac remodeling [38]. Not only animal experiments, but some clinical studies also revealed that serum levels of irisin were decreased in subjects with heart failure, especially in those with reduced ejection fraction [12, 39]. These findings show that irisin could be regarded as a promising biomarker and potential therapeutic target for cardiac injuries and remodeling.

In our present research, compared with health controls, patients with severe hypothyroidism had significantly reduced PFRs and elevated native T1 values, indicating early impaired diastolic function and diffuse myocardial lesions in the severe hypothyroidism subjects. Apart from other causes of interstitial involvement, including myocardial infarction, myocarditis, or amyloid, elevated T1 values are thought to associate closely with myocardial fibrosis, inflammation, and edema induced by hypothyroidism [40, 41]. Meanwhile, our previous studies demonstrated that myocardial involvement was common in subjects with overt hypothyroidism and elevated T1 values were relevant to cardiac function impairment, which could be significantly improved after short-term levothyroxine therapy [19, 20]. Interestingly, our ROC curve analysis showed the serum irisin exhibited significant discriminatory power for pericardial effusion in severe hypothyroidism patients, with high sensitivity and specificity. Moreover, the low-irisin group displayed distinctly higher native myocardial T1 values than the high-irisin group. However, no significant difference between the two subgroups (in the severe hypothyroidism participants) was observed in cardiac systolic and diastolic function parameters. These results might indicate that reductions in serum irisin concentrations were related to early myocardial injuries. It is noteworthy that serum irisin concentrations were independently correlated with myocardial injuries in severe hypothyroidism subjects after adjusting for age, gender, BMI, TG, TC, and FT4. Therefore, irisin may improve early diagnosis and effective treatment for myocardial injuries.

To date, it remains unknown whether the change in irisin levels is the “trigger” for myocardial damage or merely a “consequence” of myocardial damage. One possible explanation is that the lack of thyroid hormones produces overdeposition of myocardial interstitial collagens and mucopolysaccharides, leading to the development of diffuse myocardial fibrosis and edema, then following a reduction in irisin production due to long-lasting myopathy [17, 18, 42, 43].

This study has several limitations. First, our research was a cross-sectional design with a relatively small sample size, which cannot demonstrate causality. Second, exercise is considered a primary inducement of irisin secretion [44]; the influence of exercise cannot be eliminated. Nevertheless, it can certainly put forward reliable hypotheses to be confirmed and extended in future clinical and preclinical research.

In summary, low serum irisin levels may indicate diffuse myocardial injuries for patients with severe hypothyroidism. Large sample and multicenter studies are needed to further corroborate this predictive effect of irisin and provide a potential target for the prevention and treatment of myocardial involvement in hypothyroidism.

## Figures and Tables

**Table 1:** Baseline characteristics of control and severe hypothyroidism groups.

	Control group (*n* = 17)	Severe hypothyroidism group (*n* = 25)	*P* value
Age, year	33.41 ± 1.96	37.40 ± 1.92	0.167
Gender, M/F, n	2/15	3/22	1.000
BMI, kg/m^2^	22.25 ± 0.86	25.74 ± 0.70	**0.003** *∗*
HR, beats/min	70.53 ± 2.34	66.71 ± 1.90	0.210
TG, mmol/L	0.73 (0.50, 0.91)	1.44 (1.05, 2.83)	**≤ 0.001** *∗*
TC, mmol/L	4.37 ± 0.21	6.25 ± 0.31	**≤ 0.001** *∗*
HDL-C, mmol/L	1.64 ± 0.09	1.68 ± 0.10	0.813
LDL-C, mmol/L	2.47 ± 0.22	3.46 ± 0.11	**0.004** *∗*
CK, U/L	84.00 (59.00, 105.00)	242.00 (144.50, 554.00)	**≤ 0.001** *∗*
FT3, pg/mL	2.80 ± 0.11	1.59 ± 0.10	**≤ 0.001** *∗*
FT4, ng/dL	1.12 (1.03, 1.16)	0.40 (0.40, 0.44)	**≤ 0.001** *∗*
TSH, mIU/mL	1.90 (1.44, 2.81)	100.00 (96.62, 100.00)	**≤ 0.001** *∗*
Irisin, ng/mL	59.96 ± 7.14	36.38 ± 5.61	**0.014** *∗*

Data are expressed as mean ± SME or median (interquartile range) unless stated otherwise. ^*∗*^*P* < 0.05.

**Table 2:** Cardiovascular magnetic resonance parameters of control and severe hypothyroidism groups.

	Control group (*n* = 17)	Severe hypothyroidism group (*n* = 25)	*P* value
BSA, m^2^	1.65 ± 0.04	1.73 ± 0.03	0.079
EF, %	60.46 ± 1.12	60.12 ± 1.71	0.882
EDV, mL/m^2^	55.44 ± 2.70	53.65 ± 1.51	0.538
ESV, mL/m^2^	21.85 ± 1.62	21.50 ± 1.28	0.865
SV, mL/m^2^	33.68 ± 1.39	32.15 ± 0.95	0.354
CI, L/min/m^2^	2.35 ± 0.09	2.16 ± 0.08	0.133
LVMI, g/m^2^	47.80 (39.25, 54.30)	51.65 (46.95, 60.30)	0.146
PET, ms	135.00 (119.60, 149.00)	156.35 (126.98, 176.35)	0.254
PFT, ms	145.15 (110.23 158.13)	158.60 (119.55, 180.20)	0.190
PER, EDV/s	3.50 (3.20, 3.70)	3.30 (3.00, 3.80)	0.258
PFR, EDV/s	4.10 (3.45, 4.55)	3.40 (2.90, 3.90)	**0.035** *∗*
Native T1 value, ms	1063.09 ± 8.72	1192.95 ± 17.83	**≤0.001** *∗*
Pericardial effusion, n	0	10	—

Data are expressed as mean ± SME or median (interquartile range) unless stated otherwise. ^*∗*^*P* < 0.05.

**Table 3:** Cardiovascular magnetic resonance parameters of two subgroups in patients with severe hypothyroidism according to the cutoff value of serum irisin levels.

	Irisin ＜36.94 ng/mL (*n* = 14)	Irisin ≥36.94 ng/mL (*n* = 11)	*P* value
Irisin, ng/mL	15.67 ± 3.24	62.73 ± 5.53	**≤ 0.001** *∗*
BSA, m^2^	1.70 ± 0.03	1.76 ± 0.05	0.265
EF, %	59.34 ± 2.82	61.11 ± 1.62	0.618
EDV, mL/m^2^	55.46 ± 2.22	51.11 ± 1.66	0.159
ESV, mL/m^2^	22.89 ± 1.97	19.56 ± 1.19	0.204
SV, mL/m^2^	32.57 ± 1.49	31.57 ± 0.98	0.614
CI, l/min/m^2^	2.08 ± 0.10	2.27 ± 0.12	0.241
LVMI, g/m^2^	49.60 (46.48, 61.05)	54.55 (48.93, 60.75)	0.380
PET, ms	153.19 ± 7.31	149.90 ± 10.87	0.799
PFT, ms	159.09 ± 13.07	157.21 ± 15.43	0.926
Per, EDV/s	3.25 (2.95, 4.15)	3.30 (3.30, 3.60)	0.848
PFR, EDV/s	3.35 (2.75, 3.85)	3.50 (3.00, 4.10)	0.478
Native T1 value, ms	1232.91 ± 21.06	1142.09 ± 23.22	**0.008** *∗*

Data are expressed as mean ± SME or median (interquartile range) unless stated otherwise. ^*∗*^*P* < 0.05.

**Table 4:** Multivariate linear regression for the association of serum irisin levels with native T1 values in severe hypothyroidism patients.

Univariate linear regression			Multivariate linear regression^♆^			Multivariate linear regression^☧^			*P* value
Variables	*β*	95% CI	*P* value	*β*	95% CI	*P* value	*β*	95% CI	
Age	−0.886	−4.880 to 3.108	0.651	0.319	−3.494 to 4.131	0.864	−1.535	−6.206 to 3.136	0.497
Sex	13.653	−102.173 to 129.479	0.810	−9.551	−117.602 to 98.500	0.856	−51.103	−154.554 to 52.338	0.312
BMI	−7.858	−18.322 to 2.606	0.134				−6.199	−16.878 to 4.480	0.183
TC	2.602	−22.586 to 27.789	0.833				5.381	−22.632 to 33.394	0.690
TG	−6.561	−31.802 to 16.681	0.596				9.278	−15.815 to 34.371	0.328
FT3	−43.653	−115.857 to 28.554	0.224						
FT4	−655.383	−1139.062 to −171.704	**0.010** ^*∗*^				−595.752	−1094.549 to −94.956	**0.022** ^*∗*^
Irisin	−1.568	−2.760 to −0.377	**0.012** ^*∗*^	−1.617	−2.929 to −0.305	**0.018** ^*∗*^	−1.473	−2.898 to −0.047	**0.044** ^*∗*^

^♆^Model 1 adjusted for age and sex; ^☧^Model 2 further adjustment for sex, age, BMI, TG, TC, and FT4. ^*∗*^*P* < 0.05.

## Data Availability

The data generated during the current study are available from the corresponding author upon reasonable request.

## Supplementary Materials

Supplementary Materials Supplementary Table 1. Comparison of serum irisin levels and cardiovascular magnetic resonance parameters in controls and severe hypothyroidism patients with and without pericardial effusion (PE).
