# Supplementary material for: Irisin as a Potential Biomarker Associated with Myocardial Injuries in Patients with Severe Hypothyroidism
Source: Int J Endocrinol. 2021 Nov 18;2021:3116068. doi: 10.1155/2021/3116068 (PMC8616683; doi:10.1155/2021/3116068)
Supplement: Supplementary Materials — Supplementary Table 1. Comparison of serum irisin levels and cardiovascular magnetic resonance parameters in controls and severe hypothyroidism patients with and without pericardial effusion (PE). [file 3116068.f1.docx]

**Supplementary Table 1** Comparison of serum irisin levels and cardiovascular magnetic resonance parameters in controls, severe hypothyroidism patients with and without pericardial effusion (PE).

|  | Control group  (n=17) | Severe hypothyroidism patients | |
| --- | --- | --- | --- |
|  |  | Without PE (n=14) | With PE (n=10) |
| Irisin, ng/mL | 59.96 ± 7.14 | 51.85 ± 6.38 | 13.16 ± 3.93^***,^ **^##^** |
| EF, % | 60.46 ± 1.12 | 61.57 ± 1.54 | 57.95 ± 3.62 |
| EDV, ml/m^2^ | 55.44 ± 2.70 | 50.68 ± 1.48 | 57.81 ± 2.49 |
| ESV, ml/m^2^ | 21.85 ± 1.62 | 19.26 ± 1.08 | 24.65 ± 2.39 |
| SV, ml/m2 | 33.68 ± 1.39 | 31.44 ± 1.00 | 33.15 ± 1.82 |
| CI, l/min/m^2^ | 2.35 ± 0.09 | 2.20 ± 0.10 | 2.11 ± 0.44 |
| LVMI, g/ m^2^ | 47.80 (39.25, 54.30) | 52.00 (46.03, 60.10) | 51.00 (48.63, 65.35) |
| PET, ms | 135.0 (119.6, 149.0) | 154.0 (125.3, 171.4) | 159.7 (127.0, 179.7) |
| PFT, ms | 145.2 (110.2, 158.1) | 147.4 (110.0, 170.9) | 168.9 (134.7, 199.4) |
| PER, EDV/s | 3.50 (3.20, 3.70) | 3.30 (3.00, 3.80) | 3.40 (3.00, 4.70) |
| PFR, EDV/s | 4.10 (3.45, 4.55) | 3.50 (2.90, 4.10) | 3.35 (2.33, 3.68) |
| Native T1 value, ms | 1063.1 ± 8.72 | 1148.1 ± 18.69**^**^** | 1260.2 ± 21.67**^***,###^** |

Data are expressed as mean ± SME or median (interquartile range) unless stated otherwise.

**P < 0.01, ***P < 0.001 vs. the control group;

**^##^**P < 0.01, **^###^**P < 0.001 vs. the subgroup of severe hypothyroidism patients without PE.
